# Targeted Mutagenesis in the Malaria Mosquito Using TALE Nucleases

**DOI:** 10.1371/journal.pone.0074511

**Published:** 2013-08-15

**Authors:** Andrea L. Smidler, Olivier Terenzi, Julien Soichot, Elena A. Levashina, Eric Marois

**Affiliations:** 1 Institut National de la Santé et de la Recherche Médicale U963, Strasbourg, France; 2 Centre National de la Recherche Scientifique UPR9022, Strasbourg, France; Johns Hopkins University, Bloomberg School of Public Health, United States of America

## Abstract

*Anopheles gambiae*, the main mosquito vector of human malaria, is a challenging organism to manipulate genetically. As a consequence, reverse genetics studies in this disease vector have been largely limited to RNA interference experiments. Here, we report the targeted disruption of the immunity gene *TEP1* using transgenic expression of Transcription-Activator Like Effector Nucleases (TALENs), and the isolation of several *TEP1* mutant *A. gambiae* lines. These mutations inhibited protein production and rendered *TEP1* mutants hypersusceptible to *Plasmodium berghei*. The TALEN technology opens up new avenues for genetic analysis in this disease vector and may offer novel biotechnology-based approaches for malaria control.

## Introduction

Malaria is caused by *Plasmodium* parasites transmitted to their human hosts by the bite of anopheline mosquitoes. Malaria has been charged with causing more human deaths than any other disease in human history and continues to kill about 660,000 annually [1]. A reduction in the malaria death toll has been achieved thanks to vector control using insecticides and insecticide-impregnated bednets, better health care and progress in medical treatment, but this success is currently mitigated by the spread of resistance both of mosquitoes to insecticides and of *Plasmodium* to antimalarial drugs. Sequencing the genome of the main malaria vector, *Anopheles gambiae* [2], enabled the identification of hundreds of genes involved in the vector’s capacity to transmit *Plasmodium* . Altering the mosquito genome in a way that abates *Plasmodium* transmission, through transgenesis or other sophisticated genetic engineering tools, can offer new perspectives in the fight against malaria. On one hand, experimentally altering mosquito genes of interest will advance our fundamental understanding of the biological interactions between mosquito and parasite, and may help target vulnerable points in the parasite cycle. On the other hand, the prospect of releasing engineered male mosquitoes to propagate malaria resistance genes through wild susceptible vector populations has been receiving increasing attention [3,4]. Novel methods to disrupt or alter target genes of interest in the malaria mosquito would promote rapid progress towards these goals. Furthermore, targeted genetic modifications that do not require the permanent introduction of transposons in the genome are particularly desirable, as they eliminate the potential risk of subsequent unplanned transposon mobilization by natural sources of transposition factors.

Recently, a novel class of DNA-binding protein domain derived from the *Xanthomonas* Transcription Activator Like Effector (TALE) proteins [5,6] has been successfully harnessed to custom-design sequence-specific endonucleases [7,8]. These TALE nucleases (TALENs), in which the TAL DNA-binding domain is fused to the FokI endonuclease domain, are easy to engineer (e.g., [9,10]), highly predictable in their sequence specificity, and highly mutagenic [11] making them an attractive alternative to Zinc Finger Nucleases that have less predictable binding specificities and require in vitro optimization [12]. Mutations arise by imprecise repair of the TALEN-generated double-stranded breaks by the non-homologous end joining (NHEJ) repair pathway. In a number of animal species, injection of mRNA encoding TALENs have readily allowed researchers to generate mutants in their genes of interest [13–19]. Very recently, mutagenesis of an eye pigmentation gene was achieved in *Aedes* mosquitoes using this method [20]. In rice, disease resistant mutants have been produced by transgenic expression of the TALENs, whose respective transgenes were eliminated by subsequent genetic crosses once the desired mutation had been fixed [21]. We hypothesized that a similar approach would be applicable to insect vectors and set out to use TALENs to target the *TEP1* gene, a key component of the mosquito immune system.

## Results

Since the embryo microinjection procedure is technically challenging in *Anopheles gambiae* (as judged by poor survival and relatively low success rate of transgenesis in this species), we expected that mutant recovery after direct injection of TALEN-encoding plasmids or mRNA might be difficult. For this reason and to enable controlled mutagenesis experiments, we preferred transgenic expression of the TALENs in the mosquito germ cells. To obtain the proof-of-principle for gene targeting in *A. gambiae* via transgenic TALENs, we selected the well-characterized immune gene *TEP1* as a target. TEP1, a protein similar to vertebrate complement factor C3, binds *Plasmodium* parasites as they invade the mosquito intestine and kills them in a manner probably dependent on its thioester site located in the C-terminus [22–25]. *TEP1* mutants will be instrumental in further dissecting the antiparasitic complement-like system in mosquitoes.

**Figure 1:**
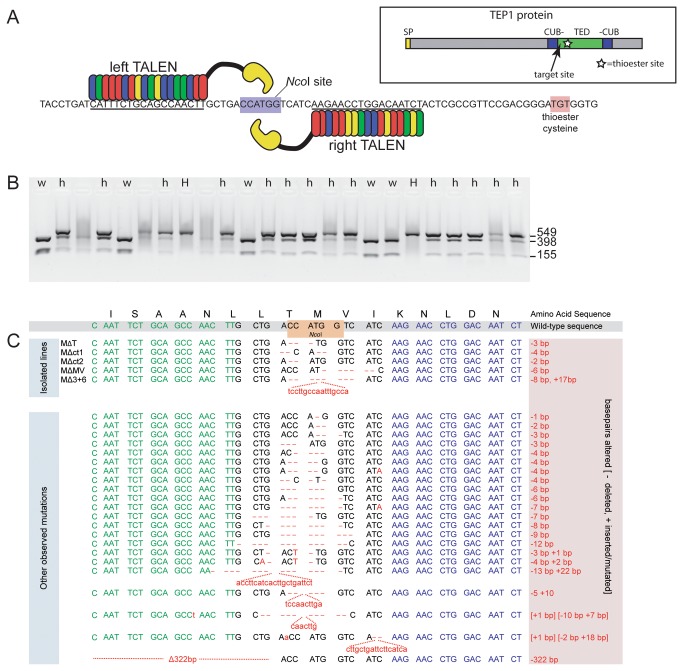
TALEN mutagenesis of the *TEP1* gene. A: Fragment from the *TEP1* gene showing the target site of the TALEN pair. Nucleotides bound by each TALEN are underlined, TALEN repeats are color-coded to show repeat/nucleotide specificity. The *Nco*I restriction site centrally located at the TALEN cleavage site is highlighted. Inset: scheme of the entire TEP1 protein showing the location of TALEN-induced mutations (SP: signal peptide; CUB: CUB domain interrupted by the TED: thioester domain; the star indicates the position of the thioester site). B: PCR assay to identify *TEP1* mutant mosquitoes. A PCR product spanning the TALEN target site is generated from individual mosquitoes (small larva or a leg from a living adult) and incubated with *Nco*I. Full cleavage of the PCR product (w) denotes a wild-type individual. Partial cleavage (h) denotes a heterozygous *TEP1* mutant. Absence of cleavage (H) corresponds to a homozygous *TEP1* mutant. C: TALEN-induced mutations in the TEP1 gene. Left and right TALEN target nucleotide sequences are shown in green and blue respectively, with the 15bp spacer sequence between the TALENs in black. The *Nco*I restriction site is highlighted in orange. Deletions are designated by a red dash or by Δ+number of missing bases. Insertions are shown in lowercase red letters. Uppercase red letters correspond to natural polymorphisms between multiple *TEP1* alleles.

TALENs function in pairs, each member of which binds a chosen 12 to 24-nucleotide sequence. The two selected target sequences are separated by 14-16 nucleotides, the optimal distance for the two *Fok*I domains of the TALENs to properly dimerize and create a double-stranded break. Cleavage of the bound DNA molecule occurs near the center of the sequence separating the two target sites. We designed a single pair of TALENs to target a site within the *TEP1* gene centered on an *Nco*I restriction site (5’-CCATGG-3’), offering the possibility to easily screen individual mosquitoes for mutations that destroyed the *Nco*I site (Figure 1A). Mutations in this region are expected to strongly affect TEP1 protein function: frame-shifts resulting in premature stop codons would remove the C-terminal third of the protein including its thioester domain, while amino-acid deletions or insertions would alter the length of the alpha-helix connecting the CUB domain to the thioester domain [26], presumably resulting in destabilization of the protein’s structure.

A distinct transgenic mosquito line was generated for each TALEN of the pair, the expression of which was driven by the *A. gambiae* Vasa promoter, active in the mosquito germline [27]. Therefore, F1 mosquitoes arising from a cross between the two lines will express both TALENs simultaneously and are expected to produce F2 gametes carrying mutations in the *TEP1* gene. To screen individual larvae within the F2 progeny, we PCR-amplified a *TEP1* fragment spanning the target site and subjected the amplification product to a restriction digest with *Nco*I (Figure 1B). Of 310 screened F2 larvae, 16 (5.16%) carried a heterozygous mutation at the target locus, as evidenced by the appearance of a PCR product that *Nco*I was unable to cleave. Thus, at least 2.58% of *TEP1* copies were mutated after exposure to one TALEN dose (i.e., one generation). This figure is a conservative estimate of mutation frequency, as we subsequently observed some mutations that left the *Nco*I recognition sequence intact. In order to obtain a mosquito population containing a higher frequency of mutations, we self-crossed successive generations of mosquitoes expressing both TALENs of the pair. This was facilitated by automated COPAS selection of larvae [28] that had inherited one copy each of the left and right TALEN genes, which are respectively associated with a red and yellow fluorescent marker expressed in the nervous system [29]. At least 1000 double-TALEN larvae were COPAS-selected and cultured for each generation. In the 7th generation (i.e., exposure to 6 TALEN mutagenic doses), 51% (49 out of 96) of the examined individual mosquitoes carried a heterozygous mutation in *TEP1*. This indicated that the frequency of mutations increased faster than predicted if mutations accumulated linearly from one generation to the next. This observation is consistent with a model where mosquitoes already carrying a heterozygous mutation employ homologous recombination to repair new TALEN-induced breaks in the wild-type chromosome, thereby effectively copying the existing mutation onto the newly damaged chromosome. This suggests that in addition to NHEJ, TALEN-caused breaks can also be repaired by homologous recombination. Therefore, the observed number of NHEJ mutations is an underestimation of the true rate of TALEN activity.

We wondered if a single TALEN of the pair is capable of causing mutations in *TEP1*. To investigate this, we sampled mosquito larvae from lines carrying a single TALEN maintained at high population levels for 8 (right TALEN) or 10 (left TALEN) generations, and again sampled larvae after about 16 generations. We expected that such a high number of generations would have allowed rare “monotalenic” mutational events to accumulate in the population. Out of 96 individual larvae tested by PCR for each sample, none carried a mutation in the *TEP1 Nco*I site. The observed absence of mutations among 576 haploid genomes exposed to single TALEN activity for 7, 9 or 15 generations suggests that single TALENs never or rarely induce mutations at their target site. To strengthen this point, we purified DNA from 2600 pooled larvae whose genomes had been exposed to one TALEN of the pair for 7 or 9 generations, and PCR-amplified the target region. PCR products appeared to be fully cleaved by *Nco*I, pointing to the absence of TALEN-induced mutations. To increase the chance of detection of a minor fraction of mutated products, we purified the region of the gel in which uncleaved PCR products may exist, cloned them into a plasmid, and examined *E. coli* transformants containing single copies of the PCR products. Again, all cloned fragments were cleaved by *Nco*I. Although we note that deep sequencing of amplicons would provide a more sensitive assay to detect rare mutations, this result further suggests that single TALENs rarely or never generate mutants. The apparent absence of single TALEN background activity was likely facilitated by our design scheme in which we used obligate heterodimeric FokI domains in TALEN construction [30,31].

**Figure 2:**
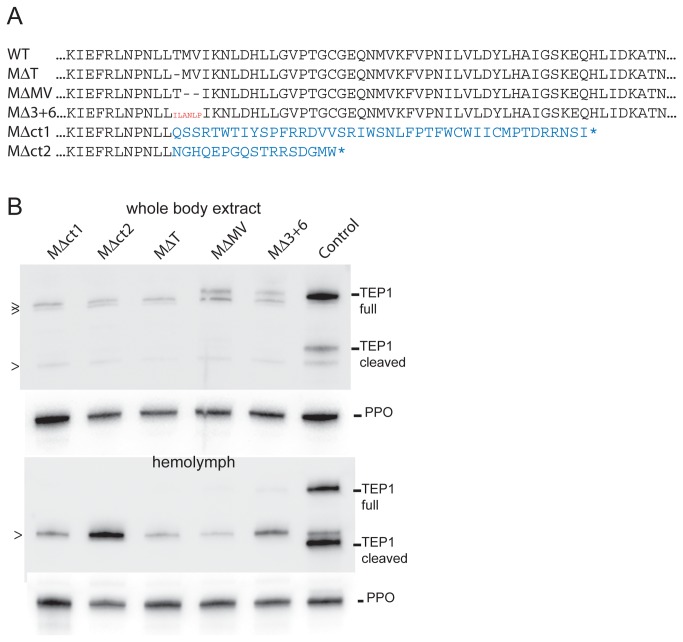
TEP1 mutant proteins. A: Fragment of the TEP1 protein encompassing the TALEN-induced mutations is shown for the wild-type (WT) and for those mutants that we maintain as homozygous mosquito lines (M^∆T^, M^∆MV^, M^∆3+6^, M^∆ct1^ and M^∆ct2^). Gaps in the protein sequence denote amino acid deletions. Inserted exogenous amino acids are shown in red. Nonsense amino acids followed by a stop codon (*), resulting from frame-shift mutations, are shown in blue. B: Immunoblots to evaluate the presence of mutant TEP1 protein in whole mosquito extract (top panels) or hemolymph. Hemolymph prophenoloxidase (PPO) serves as a loading control. In the control samples, both TEP1 full-length (full) and C-terminal fragment (cleaved) are visible. Cross-reacting background bands, some running in close proximity to TEP1 fragments, are marked on the left with ‘>’ signs.

The TALEN-generated *TEP1* mutations were very similar in nature to mutations obtained in other organisms (Figure 1C) and consisted mainly of small deletions and insertions (indels) that are the hallmark of imprecise NHEJ-mediated DNA repair. Some mutations deleted a number of nucleotides in multiples of three, resulting in the deletion of one to a few amino acids from the TEP1 protein. Other mutations introduced a frame-shift in the *TEP1* coding sequence, resulting in the loss of the entire C-terminal half of the protein, which contains features crucial for TEP1 function including the thioester domain. Using a PCR selection procedure from single legs taken from live mosquitoes, we recovered five homozygous mutant mosquito lines. These mutant lines were representative of the main classes of mutations: line M^∆T^ and M^∆MV^ are deletions of 1 (Threonine) and 2 (Methionine and Valine) amino-acids, respectively; line M^∆3+6^ lacks 3 endogenous amino-acids but gained an insertion of 6 exogenous ones, line M^∆ct1^ and M^∆ct2^ are frame-shift mutations causing the loss of the entire protein C-terminus (Figure 2A). Immunoblotting analysis failed to detect TEP1 in the Mut^∆ct1^ and M^∆ct2^ lines, while it revealed strongly reduced protein levels in M^∆T^, M^∆MV^ and M^∆3+6^ lines (Figure 2B, top). Although small amounts of TEP1 protein could be detected in whole mosquito extracts of the M^∆T,^ M^∆MV^ and M^∆3+6^ lines, these proteins did not undergo cleavage and were impaired in their secretion to the hemolymph, as no TEP1 signal was observed in immunoblotting of hemolymph samples (Figure 2B, bottom). Therefore, the alpha helix connecting the CUB and thioester domains of TEP1 [26,32] seems to be very sensitive to insertion or deletion of single amino acids that destabilize the structure and prevent proper protein synthesis and secretion.

**Figure 3:**
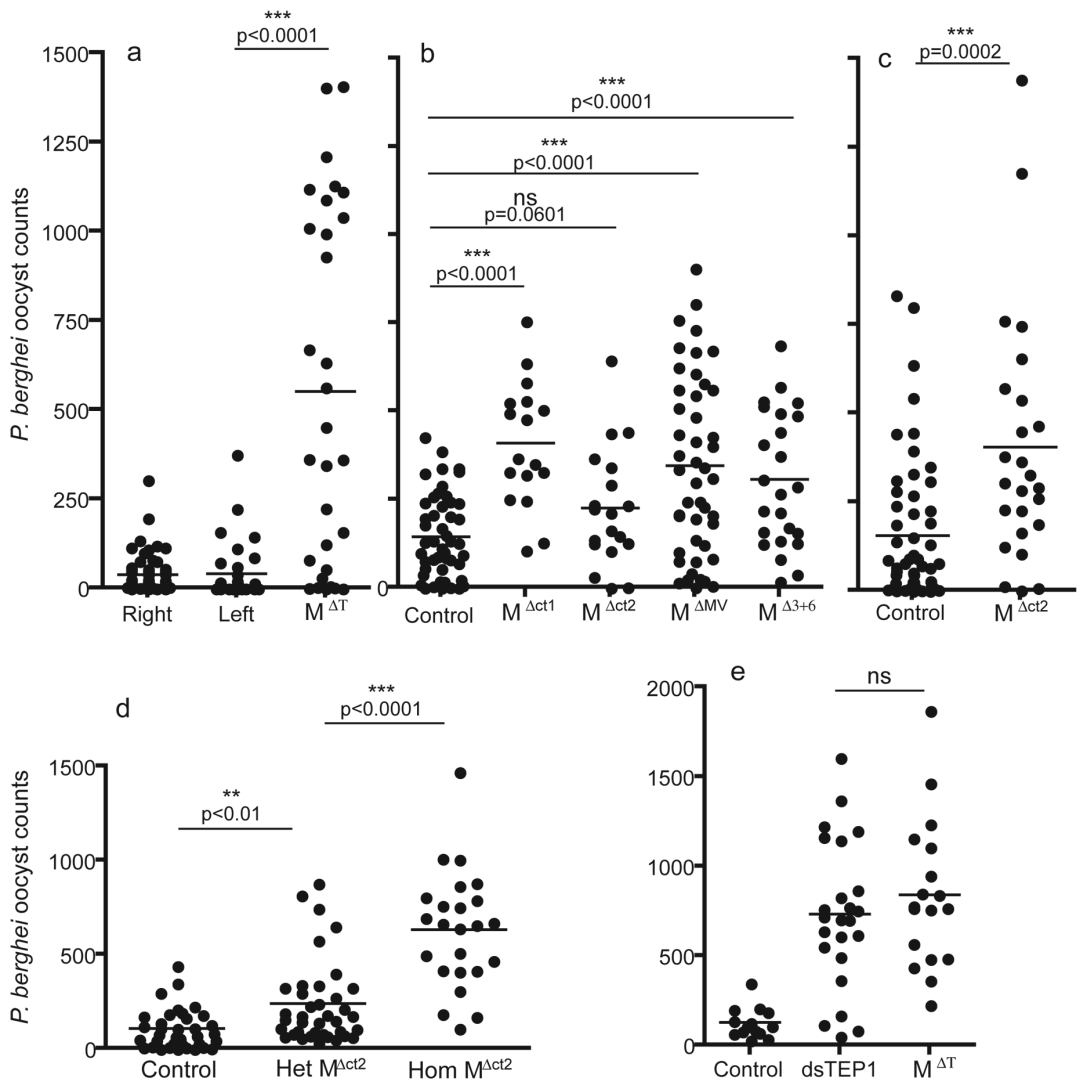
*TEP1* mutant mosquitoes are hypersusceptible to *P. berghei*. Mosquito females from five different homozygous mutant mosquito lines and from control parental lines were offered a blood meal on a *P. berghei*-infected mouse. Seven days after infection, the midgut was dissected and the number of oocysts developing in each midgut was evaluated. The statistical significance of differences in mean parasite numbers was measured with a Mann-Whitney test (mutant versus control) and with a Kruskall-Wallis test followed by Dunn’s post-test (to compare all groups in [d]). (a) M^∆T^ mosquitoes are compared to the two parental, non-mutant TALEN lines. (b) 4 different mutant lines are compared to the parental mosquito line that initially served to produce TALEN transgenic lines. This control line was verified to show the same level of susceptibility to *P. berghei* as the two TALEN daughter lines (not shown). In this experiment, three mutant lines showed significantly elevated parasite numbers compared to the control while the M^∆ct2^ line did not, presumably due to a different physiological condition of this mosquito culture. In two independent experiments (c, d), we used mosquitoes of different genotypes marked with distinct fluorescence markers and cultured the larvae together in the same water to eliminate potential confounding factors due to rearing conditions. On the day of dissection, genotypes were separated on the basis of fluorescence. The same M^∆ct2^ line shows significantly elevated parasite numbers. (d) Heterozygous M^∆ct2^ mosquitoes are compared to control and homozygous M^∆ct2^ mosquitoes: the susceptibility phenotype of the heterozygote is intermediate. (e) Control mosquitoes of the parental line, mosquitoes of the parental line injected with *TEP1* double-stranded RNA, and homozygous *TEP1* mutant mosquitoes of the M^∆T^ line are compared. TEP1 mutant and dsRNA-injected mosquitoes show comparable susceptibility to *P. berghei*.

We next assessed the phenotype of these mutant lines in comparison to the parental lines by infection assays using *Plasmodium berghei*-infected mice (Figure 3). Similar to the RNA interference knockdown phenotype of *TEP1* [22], all mutations in the homozygous state resulted in a dramatic increase in the number of developing parasites within the mosquito gut. These results confirm the pivotal role of TEP1 in antiparasitic responses. While it cannot be fully excluded that RNAi-mediated *TEP1* knockdown may also affect genes sharing some nucleotide identity with the *TEP1* sequence, such as other closely-related genes in the *TEP* family [33], mutations in *TEP1* should leave the expression of other genes unaffected.

Interestingly, while midguts from *TEP1* mutant mosquitoes often displayed impressive oocyst numbers that could exceed 1500, each experiment also yielded mutant midguts bearing no or only a few oocysts. Upon blood feeding on a single infected mouse, only well-gorged mosquito females were selected for subsequent dissection. It is therefore unlikely that some females ingested dramatically reduced numbers of *P. berghei* gametocytes. Thus, it is apparent that *TEP1*-independent mechanisms are at work to limit *Plasmodium* infection in a subset of mosquitoes.

To examine the effect of TEP1 dosage in the process of parasite killing, we compared oocyst infection levels between *TEP1* heterozygous mutant, homozygous mutant and control mosquitoes (Figure 3d). The heterozygous mutant had an intermediate susceptibility phenotype, which was closer to, but significantly different from, the control. This suggests that the efficiency of parasite killing depends on TEP1 protein levels.

The antiparasitic role of *TEP1* was discovered and characterized using RNA interference assays, in which synthetically produced double-stranded RNA homologous to a fragment of native *TEP1* is injected at a high concentration in the body of adult mosquitoes. This technique was generalized for the functional characterization of hundreds of mosquito genes [34]. However, injection per se was reported to impinge on *Plasmodium* development by the potential induction of the wounding response [35]. Therefore the mutant *TEP1* lines developed here will be useful in studies that must exclude the confounding effect of the injection procedure itself. To examine how the classical *TEP1* RNAi phenotype compares with the mutant phenotype, we compared parasite loads in M^∆T^ to ds*TEP1*-injected mosquitoes of the parental line (Figure 3e). The mutant and RNAi phenotypes were very similar, validating a posteriori the high efficiency of RNAi knockdown.

## Discussion

Here, we obtained proof of principle that TALENs can be used for targeted mutagenesis in the genome of the malaria mosquito, which is notably difficult to manipulate genetically. Recently, Aryan et al. [20] reported disruption of the eye pigmentation *kmo* gene in the dengue vector mosquito *Aedes aegypti* by injection of TALEN-encoding plasmid DNA. In the same species, successful mutagenesis of GFP and of the odorant receptor co-receptor (*orco*) was achieved by injecting mRNA encoding Zinc Finger Nucleases [36]. In both cases and in other animal systems for which TALEN mutagenesis has been reported, mutants were obtained directly upon injection of DNA or RNA into embryos. In contrast, we employed transgenically-expressed TALENs for this purpose. Besides the assurance of expressing the pair of TALENs in all germ cells, this offered the possibility to increase the frequency of mutant alleles in successive TALEN-expressing generations of mosquitoes. We disrupted the antiparasitic gene *TEP1* as a first target; future work will make use of the obtained hypersusceptible mutant lines to further dissect the role of this important anti-malarial factor in parasite killing. Beyond the research field of mosquito immunity, this study paves the way for numerous other applications such as obtaining mutations in *Anopheles* genes considered to be essential for *Plasmodium* parasite development [37–39]. Disrupting these genes, or altering specific domains on the encoded proteins, may render homozygous mutant mosquitoes unable to support parasite development. Such parasite-refractory mutant mosquitoes could be used in anti-malaria intervention schemes, including vector population replacement in endemic regions. Unless a gene-drive strategy is specifically designed to spread desired mutations, this could be achieved by the repeated release of mass-produced male mosquitoes. Where knockout mutants in a given target gene might compromise the fitness of the mosquitoes, TALEN mutagenesis offers the possibility of a gene therapy to cure mosquitoes of malaria by selecting mutations that prevent a protein’s interactions with parasite factors while preserving its other vital functions. TALEN mutagenesis could also be employed to knock out male fertility genes for use in the Sterile Insect Technique to reduce vector populations [40]. Of note, the TALEN transgenes used to obtain a desired mutation can subsequently be discarded by selection, thereby rendering the obtained homozygous mutant mosquitoes transgene-free. This could facilitate mosquito release interventions that adhere to local regulations regarding genetically modified organisms.

## Materials and Methods

### Ethics statement

Our experimental protocols were approved by Comité de Qualification Institutionnel (CQI), the ethics evaluation committee of INSERM (IRB00003888, FWA00005831). Mosquitoes were reared and blood-fed on anesthetized mice in compliance with French and European laws on animal house procedures (agreement E67-482-2 of the Direction of Veterinary services of the French Ministry of Agriculture).

### Assembling the TALENs

TALENs targeting the sites shown in Figure 1 were constructed by Golden Gate Cloning according to [9]. For TALEN assembly, we prepared two transgenesis-compatible destination vectors (annotated sequences provided in Text S1) encoding a Venus yellow fluorescent and a DsRed fluorescent transgenesis reporter gene, respectively. These vectors also contain a phage **ϕ**C31 *att*B site for genomic integration into transgenic lines harboring *att*P sites. The first module used in Golden Gate assembly provided the *Vasa* promoter characterized in [27]. The last module closed the TALEN assembly with either a FokI-DD or a FokI-RR domain [30], designed for obligate heterodimerization of the two TALENs and codon-optimized for *A. gambiae*. The annotated sequence of the three plasmids is provided in Text S1. The TALEN C and N-terminal domains flanking the repeat region were identical to those used in [7].

### A. gambiae lines and mosquito transgenesis

The TALEN-encoding vectors were inserted by **ϕ**C31 integrase-mediated transgenesis [41] into the genome of *A. gambiae* lines X1 and X13, which are derived from laboratory strain G3. These two lines carry a *PiggyBac* transgene on chromosome II, containing an *att*P docking site. Individual transgenic larvae carrying the inserted left or right TALEN genes at the X1 or X13 *att*P site were identified by their red or yellow fluorescence, respectively. Resulting adults were crossed to their non-fluorescent parental line. In the F2 generation, fluorescent homozygous larvae were COPAS-selected [28] to establish stable TALEN-expressing lines.

### Identification of TEP1 Mutants

In putative mutants (mosquitoes arising from parents expressing both TALENs), we PCR amplified a region of *TEP1* spanning the TALEN target site using Phusion or Phire Polymerases (Thermo, Fisher) and primer 5’-TCAACTTGGACATCAACAAGAAGGCCGA-3’ in combination with either 5’-GCATATCTTTGTGCCACACTTT-3’ or 5’-GCCACCGTAACGAATTTCCA-3’. The PCR product was digested with *Nco*I (Fermentas), the recognition site of which is centrally located in the sequence cut by the TALENs. PCR products corresponding to mutant *TEP1* alleles were not digested by *Nco*I. These PCR products were sequenced directly in the case of homozygous individuals, or cloned (CloneJET PCR Cloning Kit, Fermentas) and subsequently sequenced.

### Mutagenesis and mutant line recovery

For mutagenesis experiments, the two TALEN lines were crossed. From the F2 generation onwards, 1000 mosquito larvae that inherited a single copy of each TALEN were COPAS-purified to initiate the next generation. To isolate *TEP1* mutants, mosquitoes from this population were out-crossed to the parental line. The progeny carried a single TALEN and was therefore no longer subjected to TALEN mutagenesis. Among this progeny, we screened single adult mosquitoes by PCR on one leg using the Phire direct animal tissue PCR kit (Thermo, Fisher). The identified heterozygous mutants were individually crossed to the parental line. In the F2 progeny, we identified homozygous mutants by leg PCR and pooled them to start distinct homozygous mutant families.

### RNAi and infection assays

RNAi silencing of *TEP1* by double-stranded RNA injection into the thorax of adult mosquitoes and mosquito infections on mice carrying *Plasmodium berghei* GFP-con 259cl2 were performed as described [22]. To semi-automatically quantify the number of oocysts in photographs of dissected mosquito midguts, we used the “watershed segmentation” and “analyze particles” plugins of the ImageJ software after digital subtraction of the image background and smoothing of the signal. Oocyst counts obtained by this method are consistent with those obtained by manual counting of oocysts.

### Immunoblotting

Whole-body mosquito extracts were obtained by grinding one adult female mosquito in 80 µl of protein sample buffer. The sample was denatured for 3 min at 95°C and centrifuged. 8 µl were loaded on 8% SDS-polyacrylamide gels. Hemolymph samples were prepared as described [42], 10 µl of the samples were loaded. Immunoblotting was performed using standard procedures [43] with rabbit polyclonal antibodies raised against the prophenoloxidase PPO_2_ [44] or against the C-terminal half of TEP1 [45].

## Supporting Information

Text S1 **The nucleotide sequences of plasmids and building blocks used to construct the TALEN-expressing transgenesis vectors are provided.** Building blocks not listed here are published in references 5,7.
(DOCX)
