# Supplementary material for: Targeted Mutagenesis in the Malaria Mosquito Using TALE Nucleases
Source: PLoS One. 2013 Aug 15;8(8):e74511. doi: 10.1371/journal.pone.0074511 (PMC3744473; doi:10.1371/journal.pone.0074511)
Supplement: Text S1 — The nucleotide sequences of plasmids and building blocks used to construct the TALEN-expressing transgenesis vectors are provided. Building blocks not listed here are published in references 5,7. (DOCX) [file pone.0074511.s001.docx]

**Text S1**

The nucleotide sequences of plasmids and building blocks used to construct the TALEN-expressing transgenesis vectors are provided below. Building blocks not listed below are published in references [5] and [7].

**Destination vector pDSAY**

For GoldenGate assembly of a TALEN and subsequent ΦC31 integrase-mediated transgenesis, expresses a yellow fluorescent protein as transgenic selection marker.

LOCUS TALEN YFP transgenesis vector 5144 bp DNA linear 14-JUN-2013

FEATURES Location/Qualifiers

misc_feature complement(3405..3423)

/note="M13R primer"

misc_feature 1972..2021

/note="3x Pax6 binding sites"

misc_feature 2061..2068

/note="TATA box"

misc_feature 271..286

/note="M13F primer"

misc_feature 600..1174

/note="LacZ"

misc_feature 479..599

/note="lac promoter"

misc_feature 590..608

/note="M13R primer"

misc_feature 625..644

/note="T3 primer"

misc_feature 677..696

/note="SK primer"

misc_feature complement(731..747)

/note="KS primer"

misc_feature complement(800..816)

/note="M13F primer"

misc_feature complement(771..790)

/note="T7 primer"

misc_feature 1395..1621

/note="SV40 terminator"

misc_feature 1622..1898

/note="Phi C31attB site"

misc_feature 2962..3195

/note="SV40 terminator"

gene 2232..2948

/note="EYFP (Clontech)"

ORIGIN

1 CTTTCCTGCG TTATCCCCTG ATTCTGTGGA TAACCGTATT ACCGCCTTTG AGTGAGCTGA

61 TACCGCTCGC CGCAGCCGAA CGACCGAGCG CAGCGAGTCA GTGAGCGAGG AAGCGGAAGA

121 GCGCCCAATA CGCAAACCGC CTCTCCCCGC GCGTTGGCCG ATTCATTAAT GCAGCTGGCA

181 CGACAGGTTT CCCGACTGGA AAGCGGGCAG TGAGCGCAAC GCAATTAATA CGCGTACCGC

241 TAGCATGGAT GTTTTCCCAG TCACGACGTT GTAAAACGAC GGCCAGTCTT AAGCTCGGGC

301 CCCTACAGGT CACTAATACC ATCTAAGTAG TTGATTCATA GTGACTGGAT ATGTTGTGTT

361 TTACAGTATT ATGTAGTCTG TTTTTTATGC AAAATCTAAT TTAATATATT GATATTTATA

421 TCATTTTACG TTTCTCGTTC AACTTTTCTA TACAAAGTTg gtACcggatc cagagaccCG

481 CAACGCAATT AATGTGAGTT AGCTCACTCA TTAGGCACCC CAGGCTTTAC ACTTTATGCT

541 TCCGGCTCGT ATGTTGTGTG GAATTGTGAG CGGATAACAA TTTCACACAG GAAACAGCTA

601 TGACCATGAT TACGCCAAGC GCGCAATTAA CCCTCACTAA AGGGAACAAA AGCTGGAGCT

661 CCACCGCGGT GGCGGCCGCT CTAGAACTAG TGGATCCCCC GGGCTGCAGG AATTCGATAT

721 CAAGCTTATC GATACCGTCG ACCTCGAGGG GGGGCCCGGT ACCCAATTCG CCCTATAGTG

781 AGTCGTATTA CGCGCGCTCA CTGGCCGTCG TTTTACAACG TCGTGACTGG GAAAACCCTG

841 GCGTTACCCA ACTTAATCGC CTTGCAGCAC ATCCCCCTTT CGCCAGCTGG CGTAATAGCG

901 AAGAGGCCCG CACCGATCGC CCTTCCCAAC AGTTGCGCAG CCTGAATGGC GAATGGGACG

961 CGCCCTGTAG CGGCGCATTA AGCGCGGCGG GTGTGGTGGT TACGCGCAGC GTGACCGCTA

1021 CACTTGCCAG CGCCCTAGCG CCCGCTCCTT TCGCTTTCTT CCCTTCCTTT CTCGCCACGT

1081 TCGCCGGCTT TCCCCGTCAA GCTCTAAATC GGGGGCTCCC TTTAGGGTTC CGATTTAGTG

1141 CTTTACGGCA CCTCGACCCC AAAAAACTTG ATTAGGGTGA TGGTTCACGT AGTGGGCCAT

1201 CGCCCTGATA GACGGTTTTT CGCCCTTTGA CGTTGGAGTC CACGTTCTTT AATAGTGGAC

1261 TCTTGTTCCA AACTGGAACA ACACTCAACC CTATCTCGGT CTATTCTTTT GATTTATAAG

1321 GGATTTTGCC GATTTCGGCC TATTGGTTAA AAAATGAGCT GATTTAACAA AAATTTAACG

1381 CGggtctccg cttCTAGACA TAATCAGCCA TACCACATTT GTAGAGGTTT TACTTGCTTT

1441 AAAAAACCTC CCACACCTCC CCCTGAACCT GAAACATAAA ATGAATGCAA TTGTTGTTGT

1501 TAACTTGTTT ATTGCAGCTT ATAATGGTTA CAAATAAAGC AATAGCATCA CAAATTTCAC

1561 AAATAAAGCA TTTTTCTTCA CTGCATTCTA GTTGTGGTTT GTCCAAACTC ATCAATGTAT

1621 CtcgaCGATG TAGGTCACaG TCTCGAAGCC GCGGTGCGGG TGCCAGGGCG TGCCCTTGGG

1681 CTCCCCGGGC GCGTACTCCA CCTCACCCAT CTGGTCCATC ATGATGAACG GGTCGAGGTG

1741 GCGGTAGTTG ATCCCGGCGA ACGCGCGGCG CACCGGGAAG CCCTCGCCCT CGAAACCGCT

1801 GGGCGCGGTG GTCACGGTGA GCACGGGACG TGCGACGGCG TCGGCGGGTG CGGATACGCG

1861 GGGCAGCGTC AGCGGGTTCT CGACGGTCAC GGCGGGCAat tCCTGCAGAC TTCCGGTATC

1921 TCGCGTTTGT TTGATCGCAC GGTTCCCACA ATGGTTAATT CGAGCTCGCC CGGGGATCTA

1981 ATTCAATTAG AGACTAATTC AATTAGAGCT AATTCAATTA GGATCCAAGC TTATCGATTT

2041 CGAACCCTCG ACCGCCGGAG TATAAATAGA GGCGCTTCGT CTACGGAGCG ACAATTCAAT

2101 TCAAACAAGC AAAGTGAACA CGTCGCTAAG CGAAAGCTAA GCAAATAAAC AAGCGCAGCT

2161 GAACAAGCTA AACAATCGGG GTACCGCTAG AGTCGACGGT ACCGCGGGCC CGGGATCCAC

2221 CGGTCGCCAC CATGGTGAGC AAGGGCGAGG AGCTGTTCAC CGGGGTGGTG CCCATCCTGG

2281 TCGAGCTGGA CGGCGACGTA AACGGCCACA AGTTCAGCGT GTCCGGCGAG GGCGAGGGCG

2341 ATGCCACCTA CGGCAAGCTG ACCCTGAAGT TCATCTGCAC CACCGGCAAG CTGCCCGTGC

2401 CCTGGCCCAC CCTCGTGACC ACCTTCGGCT ACGGCCTGCA GTGCTTCGCC CGCTACCCCG

2461 ACCACATGAA GCAGCACGAC TTCTTCAAGT CCGCCATGCC CGAAGGCTAC GTCCAGGAGC

2521 GCACCATCTT CTTCAAGGAC GACGGCAACT ACAAGACCCG CGCCGAGGTG AAGTTCGAGG

2581 GCGACACCCT GGTGAACCGC ATCGAGCTGA AGGGCATCGA CTTCAAGGAG GACGGCAACA

2641 TCCTGGGGCA CAAGCTGGAG TACAACTACA ACAGCCACAA CGTCTATATC ATGGCCGACA

2701 AGCAGAAGAA CGGCATCAAG GTGAACTTCA AGATCCGCCA CAACATCGAG GACGGCAGCG

2761 TGCAGCTCGC CGACCACTAC CAGCAGAACA CCCCCATCGG CGACGGCCCC GTGCTGCTGC

2821 CCGACAACCA CTACCTGAGC TACCAGTCCG CCCTGAGCAA AGACCCCAAC GAGAAGCGCG

2881 ATCACATGGT CCTGCTGGAG TTCGTGACCG CCGCCGGGAT CACTCTCGGC ATGGACGAGC

2941 TGTACAAGTA AAGCGGCCGC GACTCTAGAT CATAATCAGC CATACCACAT TTGTAGAGGT

3001 TTTACTTGCT TTAAAAAACC TCCCACACCT CCCCCTGAAC CTGAAACATA AAATGAATGC

3061 AATTGTTGTT GTTAACTTGT TTATTGCAGC TTATAATGGT TACAAATAAA GCAATAGCAT

3121 CACAAATTTC ACAAATAAAG CATTTTTTTC ACTGCATTCT AGTTGTGGTT TGTCCAAACT

3181 CATCAATGTA TCTTAAAGCT TATCGATACG CGTACGGCGC GCCTAGAGCG GCCGCCACCG

3241 CGGTGGAGCT CGAGTACCCA GCTTTCTTGT ACAAAGTTGG CATTATAAGA AAGCATTGCT

3301 TATCAATTTG TTGCAACGAA CAGGTCACTA TCAGTCAAAA TAAAATCATT ATTTGCCATC

3361 CAGCTGCAGG GCGGCCGCGA TATCCCCTAT AGTGAGTCGT ATTACATGGT CATAGCTGTT

3421 TCCTGGCAGC TCTGGCCCGT GTCTCAAAAT CTCTGATGTT ACATTGCACA AGATAAAAAT

3481 ATATCATCAT GAACAATAAA ACTGTCTGCT TACATAAACA GTAATACAAG GGGTGTTATG

3541 AGCCATATTC AACGGGAAAC GTCGAGGCCG CGATTAAATT CCAACATGGA TGCTGATTTA

3601 TATGGGTATA AATGGGCTCG CGATAATGTC GGGCAATCAG GTGCGACAAT CTATCGCTTG

3661 TATGGGAAGC CCGATGCGCC AGAGTTGTTT CTGAAACATG GCAAAGGTAG CGTTGCCAAT

3721 GATGTTACAG ATGAGATGGT CAGACTAAAC TGGCTGACGG AATTTATGCC TCTTCCGACC

3781 ATCAAGCATT TTATCCGTAC TCCTGATGAT GCATGGTTAC TCACCACTGC GATCCCCGGA

3841 AAAACAGCAT TCCAGGTATT AGAAGAATAT CCTGATTCAG GTGAAAATAT TGTTGATGCG

3901 CTGGCAGTGT TCCTGCGCCG GTTGCATTCG ATTCCTGTTT GTAATTGTCC TTTTAACAGC

3961 GATCGCGTAT TTCGTCTCGC TCAGGCGCAA TCACGAATGA ATAACGGTTT GGTTGATGCG

4021 AGTGATTTTG ATGACGAGCG TAATGGCTGG CCTGTTGAAC AAGTCTGGAA AGAAATGCAT

4081 AAACTTTTGC CATTCTCACC GGATTCAGTC GTCACTCATG GTGATTTCTC ACTTGATAAC

4141 CTTATTTTTG ACGAGGGGAA ATTAATAGGT TGTATTGATG TTGGACGAGT CGGAATCGCA

4201 GACCGATACC AGGATCTTGC CATCCTATGG AACTGCCTCG GTGAGTTTTC TCCTTCATTA

4261 CAGAAACGGC TTTTTCAAAA ATATGGTATT GATAATCCTG ATATGAATAA ATTGCAGTTT

4321 CATTTGATGC TCGATGAGTT TTTCTAATCA GAATTGGTTA ATTGGTTGTA ACACTGGCAG

4381 AGCATTACGC TGACTTGACG GGACGGCGCA AGCTCATGAC CAAAATCCCT TAACGTGAGT

4441 TACGCGTCGT TCCACTGAGC GTCAGACCCC GTAGAAAAGA TCAAAGGATC TTCTTGAGAT

4501 CCTTTTTTTC TGCGCGTAAT CTGCTGCTTG CAAACAAAAA AACCACCGCT ACCAGCGGTG

4561 GTTTGTTTGC CGGATCAAGA GCTACCAACT CTTTTTCCGA AGGTAACTGG CTTCAGCAGA

4621 GCGCAGATAC CAAATACTGT TCTTCTAGTG TAGCCGTAGT TAGGCCACCA CTTCAAGAAC

4681 TCTGTAGCAC CGCCTACATA CCTCGCTCTG CTAATCCTGT TACCAGTGGC TGCTGCCAGT

4741 GGCGATAAGT CGTGTCTTAC CGGGTTGGAC TCAAGACGAT AGTTACCGGA TAAGGCGCAG

4801 CGGTCGGGCT GAACGGGGGG TTCGTGCACA CAGCCCAGCT TGGAGCGAAC GACCTACACC

4861 GAACTGAGAT ACCTACAGCG TGAGCTATGA GAAAGCGCCA CGCTTCCCGA AGGGAGAAAG

4921 GCGGACAGGT ATCCGGTAAG CGGCAGGGTC GGAACAGGAG AGCGCACGAG GGAGCTTCCA

4981 GGGGGAAACG CCTGGTATCT TTATAGTCCT GTCGGGTTTC GCCACCTCTG ACTTGAGCGT

5041 CGATTTTTGT GATGCTCGTC AGGGGGGCGG AGCCTATGGA AAAACGCCAG CAACGCGGCC

5101 TTTTTACGGT TCCTGGCCTT TTGCTGGCCT TTTGCTCACA TGTT

//

**Destination vector pDSAR**

For GoldenGate assembly of a TALEN and subsequent ΦC31 integrase-mediated transgenesis, expresses a red fluorescent protein as transgenic selection marker.

LOCUS TALEN RFP transgenesis vector 5103 bp DNA linear 14-JUN-2013

FEATURES Location/Qualifiers

misc_feature complement(3364..3382)

/note="M13R primer"

misc_feature 2231..2912

/note="DsRed"

misc_feature 1972..2021

/note="3x Pax6 binding sites"

misc_feature 2061..2068

/note="TATA box"

misc_feature 271..286

/note="M13F primer"

misc_feature 600..1174

/note="LacZ"

misc_feature 479..599

/note="lac promoter"

misc_feature 590..608

/note="M13R primer"

misc_feature 625..644

/note="T3 primer"

misc_feature 677..696

/note="SK primer"

misc_feature complement(731..747)

/note="KS primer"

misc_feature complement(800..816)

/note="M13F primer"

misc_feature complement(771..790)

/note="T7 primer"

misc_feature 1395..1621

/note="SV40 terminator"

misc_feature 1622..1898

/note="Phi C31attB site"

misc_feature 2914..3154

/note="SV40 terminator"

ORIGIN

1 CTTTCCTGCG TTATCCCCTG ATTCTGTGGA TAACCGTATT ACCGCCTTTG AGTGAGCTGA

61 TACCGCTCGC CGCAGCCGAA CGACCGAGCG CAGCGAGTCA GTGAGCGAGG AAGCGGAAGA

121 GCGCCCAATA CGCAAACCGC CTCTCCCCGC GCGTTGGCCG ATTCATTAAT GCAGCTGGCA

181 CGACAGGTTT CCCGACTGGA AAGCGGGCAG TGAGCGCAAC GCAATTAATA CGCGTACCGC

241 TAGCATGGAT GTTTTCCCAG TCACGACGTT GTAAAACGAC GGCCAGTCTT AAGCTCGGGC

301 CCCTACAGGT CACTAATACC ATCTAAGTAG TTGATTCATA GTGACTGGAT ATGTTGTGTT

361 TTACAGTATT ATGTAGTCTG TTTTTTATGC AAAATCTAAT TTAATATATT GATATTTATA

421 TCATTTTACG TTTCTCGTTC AACTTTTCTA TACAAAGTTg gtACcggatc cagagaccCG

481 CAACGCAATT AATGTGAGTT AGCTCACTCA TTAGGCACCC CAGGCTTTAC ACTTTATGCT

541 TCCGGCTCGT ATGTTGTGTG GAATTGTGAG CGGATAACAA TTTCACACAG GAAACAGCTA

601 TGACCATGAT TACGCCAAGC GCGCAATTAA CCCTCACTAA AGGGAACAAA AGCTGGAGCT

661 CCACCGCGGT GGCGGCCGCT CTAGAACTAG TGGATCCCCC GGGCTGCAGG AATTCGATAT

721 CAAGCTTATC GATACCGTCG ACCTCGAGGG GGGGCCCGGT ACCCAATTCG CCCTATAGTG

781 AGTCGTATTA CGCGCGCTCA CTGGCCGTCG TTTTACAACG TCGTGACTGG GAAAACCCTG

841 GCGTTACCCA ACTTAATCGC CTTGCAGCAC ATCCCCCTTT CGCCAGCTGG CGTAATAGCG

901 AAGAGGCCCG CACCGATCGC CCTTCCCAAC AGTTGCGCAG CCTGAATGGC GAATGGGACG

961 CGCCCTGTAG CGGCGCATTA AGCGCGGCGG GTGTGGTGGT TACGCGCAGC GTGACCGCTA

1021 CACTTGCCAG CGCCCTAGCG CCCGCTCCTT TCGCTTTCTT CCCTTCCTTT CTCGCCACGT

1081 TCGCCGGCTT TCCCCGTCAA GCTCTAAATC GGGGGCTCCC TTTAGGGTTC CGATTTAGTG

1141 CTTTACGGCA CCTCGACCCC AAAAAACTTG ATTAGGGTGA TGGTTCACGT AGTGGGCCAT

1201 CGCCCTGATA GACGGTTTTT CGCCCTTTGA CGTTGGAGTC CACGTTCTTT AATAGTGGAC

1261 TCTTGTTCCA AACTGGAACA ACACTCAACC CTATCTCGGT CTATTCTTTT GATTTATAAG

1321 GGATTTTGCC GATTTCGGCC TATTGGTTAA AAAATGAGCT GATTTAACAA AAATTTAACG

1381 CGggtctccg cttCTAGACA TAATCAGCCA TACCACATTT GTAGAGGTTT TACTTGCTTT

1441 AAAAAACCTC CCACACCTCC CCCTGAACCT GAAACATAAA ATGAATGCAA TTGTTGTTGT

1501 TAACTTGTTT ATTGCAGCTT ATAATGGTTA CAAATAAAGC AATAGCATCA CAAATTTCAC

1561 AAATAAAGCA TTTTTCTTCA CTGCATTCTA GTTGTGGTTT GTCCAAACTC ATCAATGTAT

1621 CtcgaCGATG TAGGTCACaG TCTCGAAGCC GCGGTGCGGG TGCCAGGGCG TGCCCTTGGG

1681 CTCCCCGGGC GCGTACTCCA CCTCACCCAT CTGGTCCATC ATGATGAACG GGTCGAGGTG

1741 GCGGTAGTTG ATCCCGGCGA ACGCGCGGCG CACCGGGAAG CCCTCGCCCT CGAAACCGCT

1801 GGGCGCGGTG GTCACGGTGA GCACGGGACG TGCGACGGCG TCGGCGGGTG CGGATACGCG

1861 GGGCAGCGTC AGCGGGTTCT CGACGGTCAC GGCGGGCAat tCCTGCAGAC TTCCGGTATC

1921 TCGCGTTTGT TTGATCGCAC GGTTCCCACA ATGGTTAATT CGAGCTCGCC CGGGGATCTA

1981 ATTCAATTAG AGACTAATTC AATTAGAGCT AATTCAATTA GGATCCAAGC TTATCGATTT

2041 CGAACCCTCG ACCGCCGGAG TATAAATAGA GGCGCTTCGT CTACGGAGCG ACAATTCAAT

2101 TCAAACAAGC AAAGTGAACA CGTCGCTAAG CGAAAGCTAA GCAAATAAAC AAGCGCAGCT

2161 GAACAAGCTA AACAATCGGG GTACCGCTAG AGTCGACGGT ACCGCGGGCC CGGGATCCAC

2221 CGGTCGCCAC CATGGTGCGC TCCTCCAAGA ACGTCATCAA GGAGTTCATG CGCTTCAAGG

2281 TGCGCATGGA GGGCACCGTG AACGGCCACG AGTTCGAGAT CGAGGGCGAG GGCGAGGGCC

2341 GCCCCTACGA GGGCCACAAC ACCGTGAAGC TGAAGGTGAC CAAGGGCGGC CCCCTGCCCT

2401 TCGCCTGGGA CATCCTGTCC CCCCAGTTCC AGTACGGCTC CAAGGTGTAC GTGAAGCACC

2461 CCGCCGACAT CCCCGACTAC AAGAAGCTGT CCTTCCCCGA GGGCTTCAAG TGGGAGCGCG

2521 TGATGAACTT CGAGGACGGC GGCGTGGTGA CCGTGACCCA GGACTCCTCC CTGCAGGACG

2581 GCTGCTTCAT CTACAAGGTG AAGTTCATCG GCGTGAACTT CCCCTCCGAC GGCCCCGTAA

2641 TGCAGAAGAA GACCATGGGC TGGGAGGCCT CCACCGAGCG CCTGTACCCC CGCGACGGCG

2701 TGCTGAAGGG CGAGATCCAC AAGGCCCTGA AGCTGAAGGA CGGCGGCCAC TACCTGGTGG

2761 AGTTCAAGTC CATCTACATG GCCAAGAAGC CCGTGCAGCT GCCCGGCTAC TACTACGTGG

2821 ACTCCAAGCT GGACATCACC TCCCACAACG AGGACTACAC CATCGTGGAg CAGTACGAGC

2881 GCACCGAGGG CCGCCACCAC CTGTTCCTGT AGCGGCCGCG ACTCTAGATC ATAATCAGCC

2941 ATACCACATT TGTAGAGGTT TTACTTGCTT TAAAAAACCT CCCACACCTC CCCCTGAACC

3001 TGAAACATAA AATGAATGCA ATTGTTGTTG TTAACTTGTT TATTGCAGCT TATAATGGTT

3061 ACAAATAAAG CAATAGCATC ACAAATTTCA CAAATAAAGC ATTTTTTTCA CTGCATTCTA

3121 GTTGTGGTTT GTCCAAACTC ATCAATGTAT CTTAAAGCTT ATCGATACGC GTACGGCGCG

3181 CCTAGAGCGG CCGCCACCGC GGTGGAGCTC GAGTACCCAG CTTTCTTGTA CAAAGTTGGC

3241 ATTATAAGAA AGCATTGCTT ATCAATTTGT TGCAACGAAC AGGTCACTAT CAGTCAAAAT

3301 AAAATCATTA TTTGCCATCC AGCTGCAGGG CGGCCGCGAT ATCCCCTATA GTGAGTCGTA

3361 TTACATGGTC ATAGCTGTTT CCTGGCAGCT CTGGCCCGTG TCTCAAAATC TCTGATGTTA

3421 CATTGCACAA GATAAAAATA TATCATCATG AACAATAAAA CTGTCTGCTT ACATAAACAG

3481 TAATACAAGG GGTGTTATGA GCCATATTCA ACGGGAAACG TCGAGGCCGC GATTAAATTC

3541 CAACATGGAT GCTGATTTAT ATGGGTATAA ATGGGCTCGC GATAATGTCG GGCAATCAGG

3601 TGCGACAATC TATCGCTTGT ATGGGAAGCC CGATGCGCCA GAGTTGTTTC TGAAACATGG

3661 CAAAGGTAGC GTTGCCAATG ATGTTACAGA TGAGATGGTC AGACTAAACT GGCTGACGGA

3721 ATTTATGCCT CTTCCGACCA TCAAGCATTT TATCCGTACT CCTGATGATG CATGGTTACT

3781 CACCACTGCG ATCCCCGGAA AAACAGCATT CCAGGTATTA GAAGAATATC CTGATTCAGG

3841 TGAAAATATT GTTGATGCGC TGGCAGTGTT CCTGCGCCGG TTGCATTCGA TTCCTGTTTG

3901 TAATTGTCCT TTTAACAGCG ATCGCGTATT TCGTCTCGCT CAGGCGCAAT CACGAATGAA

3961 TAACGGTTTG GTTGATGCGA GTGATTTTGA TGACGAGCGT AATGGCTGGC CTGTTGAACA

4021 AGTCTGGAAA GAAATGCATA AACTTTTGCC ATTCTCACCG GATTCAGTCG TCACTCATGG

4081 TGATTTCTCA CTTGATAACC TTATTTTTGA CGAGGGGAAA TTAATAGGTT GTATTGATGT

4141 TGGACGAGTC GGAATCGCAG ACCGATACCA GGATCTTGCC ATCCTATGGA ACTGCCTCGG

4201 TGAGTTTTCT CCTTCATTAC AGAAACGGCT TTTTCAAAAA TATGGTATTG ATAATCCTGA

4261 TATGAATAAA TTGCAGTTTC ATTTGATGCT CGATGAGTTT TTCTAATCAG AATTGGTTAA

4321 TTGGTTGTAA CACTGGCAGA GCATTACGCT GACTTGACGG GACGGCGCAA GCTCATGACC

4381 AAAATCCCTT AACGTGAGTT ACGCGTCGTT CCACTGAGCG TCAGACCCCG TAGAAAAGAT

4441 CAAAGGATCT TCTTGAGATC CTTTTTTTCT GCGCGTAATC TGCTGCTTGC AAACAAAAAA

4501 ACCACCGCTA CCAGCGGTGG TTTGTTTGCC GGATCAAGAG CTACCAACTC TTTTTCCGAA

4561 GGTAACTGGC TTCAGCAGAG CGCAGATACC AAATACTGTT CTTCTAGTGT AGCCGTAGTT

4621 AGGCCACCAC TTCAAGAACT CTGTAGCACC GCCTACATAC CTCGCTCTGC TAATCCTGTT

4681 ACCAGTGGCT GCTGCCAGTG GCGATAAGTC GTGTCTTACC GGGTTGGACT CAAGACGATA

4741 GTTACCGGAT AAGGCGCAGC GGTCGGGCTG AACGGGGGGT TCGTGCACAC AGCCCAGCTT

4801 GGAGCGAACG ACCTACACCG AACTGAGATA CCTACAGCGT GAGCTATGAG AAAGCGCCAC

4861 GCTTCCCGAA GGGAGAAAGG CGGACAGGTA TCCGGTAAGC GGCAGGGTCG GAACAGGAGA

4921 GCGCACGAGG GAGCTTCCAG GGGGAAACGC CTGGTATCTT TATAGTCCTG TCGGGTTTCG

4981 CCACCTCTGA CTTGAGCGTC GATTTTTGTG ATGCTCGTCA GGGGGGCGGA GCCTATGGAA

5041 AAACGCCAGC AACGCGGCCT TTTTACGGTT CCTGGCCTTT TGCTGGCCTT TTGCTCACAT

5101 GTT

//

***Fok*I domain module for GoldenGate cloning, DD mutant, codon-optimized for *Anopheles gambiae.***

Shown is the synthetic *Fok*I domain sequence flanked by *Bsa*I sites cloned in pUC57.

LOCUS DD-AgOptFokEndDom 628 bp DNA linear 09-AUG-2011

FEATURES Location/Qualifiers

misc_feature 20..607

/note="FokI nuclease domain"

misc_feature 317..331

/note="DD"

ORIGIN

1 gatatcGGTC TCTGGTGCAC AGCTGGTCAA GTCGGAACTG GAGGAGAAGA AAAGCGAGCT

61 CCGGCACAAG CTCAAGTATG TGCCCCATGA ATACATCGAA TTGATTGAGA TCGCACGAAA

121 TTCGACCCAA GACCGTATCC TTGAGATGAA GGTGATGGAA TTTTTCATGA AGGTGTACGG

181 TTACCGTGGA AAGCACCTAG GCGGCTCTCG CAAGCCAGAT GGAGCTATAT ACACCGTGGG

241 TAGCCCCATT GATTACGGTG TTATCGTCGA CACCAAGGCG TACTCCGGCG GATACAACCT

301 CCCGATCGGC CAGGCGGACG AAATGCAGga CTACGTAGAG GAGAATCAGA CGCGTAACAA

361 GCACATCAAC CCGAACGAGT GGTGGAAGGT GTACCCGAGC AGTGTCACGG AGTTCAAGTT

421 CCTGTTCGTC TCGGGGCACT TCAAGGGCAA CTACAAGGCC CAGCTGACGC GCCTGAACCA

481 CATCACGAAC TGCAACGGCG CCGTCCTGTC GGTGGAGGAG CTGCTGATCG GCGGCGAGAT

541 GATCAAGGCC GGaACCCTGA CTCTGGAGGA GGTGCGCCGC AAGTTCAACA ACGGAGAGAT

601 CAACTTCTAA AGCTTAGAGA CCgatatc

//

***Fok*I domain module for GoldenGate cloning, RR mutant, codon-optimized for *Anopheles gambiae.***

Shown is the synthetic *Fok*I domain sequence flanked by *Bsa*I sites cloned in pUC57.

LOCUS NatFokEndDom 628 bp DNA linear 09-AUG-2011

FEATURES Location/Qualifiers

misc_feature 20..607

/note="FokI nuclease domain"

misc_feature 317..331

/note="RR"

ORIGIN

1 gatatcGGTC TCTGGTGCAC AGCTGGTCAA GTCGGAACTG GAGGAGAAGA AAAGCGAGCT

61 CCGGCACAAG CTCAAGTATG TGCCCCATGA ATACATCGAA TTGATTGAGA TCGCACGAAA

121 TTCGACCCAA GACCGTATCC TTGAGATGAA GGTGATGGAA TTTTTCATGA AGGTGTACGG

181 TTACCGTGGA AAGCACCTAG GCGGCTCTCG CAAGCCAGAT GGAGCTATAT ACACCGTGGG

241 TAGCCCCATT GATTACGGTG TTATCGTCGA CACCAAGGCG TACTCCGGCG GATACAACCT

301 CCCGATCGGC CAGGCGcgCG AAATGCAGCG CTACGTAGAG GAGAATCAGA CGCGTAACAA

361 GCACATCAAC CCGAACGAGT GGTGGAAGGT GTACCCGAGC AGTGTCACGG AGTTCAAGTT

421 CCTGTTCGTC TCGGGGCACT TCAAGGGCAA CTACAAGGCC CAGCTGACGC GCCTGAACCA

481 CATCACGAAC TGCAACGGCG CCGTCCTGTC GGTGGAGGAG CTGCTGATCG GCGGCGAGAT

541 GATCAAGGCC GGaACCCTGA CTCTGGAGGA GGTGCGCCGC AAGTTCAACA ACGGAGAGAT

601 CAACTTCTAA AGCTTAGAGA CCgatatc

//

***Anopheles gambiae Vasa* promoter module for GoldenGate cloning.**

Shown is the *Vasa* promoter sequence flanked by *Bsa*I sites cloned in pUC57.

LOCUS Ag vasa for GGC 2319 bp DNA linear 04-MAR-2013

FEATURES Location/Qualifiers

misc_feature 13..2305

/dnas_title="5'Vas"

/vntifkey="21"

/label=5'Vas

ORIGIN

1 CggtctcaAT CCcgatgtag aacgcgagca aattcttttc cttccatgac agcagcagct

61 acagtgggaa gccgaacgtc agacgtgttt gacatgccga actgggcggg aaaattacag

121 cgtgcgcttt gttttcaagc aaatcacaac tcgctgcaaa caaaaccgtt gagaaattga

181 ttgttttata atttgtattg tattttattt gttataataa actaaaaaga catacttttt

241 gcatatttta tacataaaaa catacatgca gcattataaa acacatataa accctccctg

301 tagagtcccg tatcgaaatc ttccatccta gttgcacagt acgacggacg agtaggccgt

361 gtccgtgcaa attccagctt ttagcagtct tttgctcgga gcactcgcgg cgagtcggag

421 gtttctgctg aggtgcttag cgctaaatta gccaattgct tttgcaagtg aaataaccag

481 ccgaatagta cttcaaaact caggtaagtg aactagtttt atagaacaaa tgtttgtttg

541 ttagaagtta gtgaagtgtt tgtgaaaaaa atctctcatt tcggcaaaac taacgtaact

601 gatttcaaat tgaattattg ttttgtgatg ttatattatt tcatccagtt gattagtatt

661 ttcttagtta tgttcaaaat acagttaaat taaatttcat ttcatttact cataaaataa

721 tctcttggct tatttaattt ttctcgaatt cgcttgtatt gttcagtagc acgcgccatt

781 cgccctttgt ttcattttgt acctgctccc actaacacac tggcagtgcg aaacaaaagc

841 cttcgcacgc gttgctggta ttagagtgtg tgcgtgtgtg tgttgagcgc tctgtcaaaa

901 tcggctgttg ccgccggtac cgaaattgcc tgttcgcacg ctgttcgtaa acattccgtg

961 gtgtgtatcg tgtgttgtgc atgttgcgcg cctcccccct tttgatagca ggctgccgtg

1021 gctgccgtgg tgtgtggcgc agttgagttt ttggattaat tttctaagga aatggcacga

1081 gaagagcggt ggcagtgtgt tggtttgctc tgtcccttcc tttctgtgtg aagtgttctt

1141 acagcacagc acgtatccac caccgcacac agagcaggca aggaagtgga agtgaacaag

1201 tgtgctgcgc atgcatgtgt gtggggggca ttttagctga gatcgtcgtt atttgagaag

1261 cggtataggg gccagtcggt gtcgacgtac ggaagcggtt tagttttaat ccaagcgtat

1321 cccgtcgtgg agtggttgtg tggctctgtg tgctctcata tcagttccag agtgaggtta

1381 gtagaatcac agtccttggc ctttttcgtt acaagatatc cagaaggatg gcgttatttc

1441 cacagcttac catggtgctc ttgtttgctc gaatcagggg agaaaaacag tttcgtgttt

1501 catgaaccgc agttggcact ggagcggatt caaaagtctt cgatatgcaa tagataagag

1561 agtcgttggg gcatagttgg gaagcctttc cgagatgtgg agtttccgag aggagaaatg

1621 gtgctttcgt gcacgttccg ggacagcggg ccccgcgaag agcatctcgt tgtcgttcat

1681 ccggcaataa ttgatgcgaa aagcgcgcgc gccactggct tagcgcagtg tacacagtga

1741 tattcaccta cacacacaga ggcacacgcc ttcacacgcg cgcgtgcttc aaaggctact

1801 tcggtggcgg tgtgtgaggt cgcttgcaat ggacaatgaa aatttcgctg gaaaatacca

1861 tcgtctcttt aggttgcaat gggtgcgggt agagcggtgg tcgtcgatat tggtggtgta

1921 gtgtgtgtgt gtgtgtgtgt gtgtgtgtgt gtgtgtgtgt gtgtgtgtgt gtgtgtgtgt

1981 gtgtgtgtgt gtgtgtgtgt gtgtgtgtgt gtgtgtgcaa cggcaattat tttttgtaat

2041 atttcgacca tctttctttc tctctctcca cgtgctgctg ctgttgctgc tgctgctgca

2101 ttgcatgttc cactattcct ctcggtttgt gcctgcggac gccattgcta gtcgaaagag

2161 agtcgccgtt agtcgcgctt cgagcaacgg acacgttttt tggttgaaac caacagcttt

2221 tttcatcttc gggagacaca cagatctcga atcgtacatt cccataagga gaattgtcat

2281 cttccggtga ataaagaaag gaaacAAtat gTGAGACCC

//
